# Bibliometric Analysis of Global Research on Cancer Photodynamic Therapy: Focus on Nano-Related Research

**DOI:** 10.3389/fphar.2022.927219

**Published:** 2022-06-16

**Authors:** Kunming Cheng, Qiang Guo, Zefeng Shen, Weiguang Yang, Yulin Wang, Zaijie Sun, Haiyang Wu

**Affiliations:** ^1^ Department of Intensive Care Unit, The Second Affiliated Hospital of Zhengzhou University, Zhengzhou, China; ^2^ Department of Orthopaedics, Baodi Clinical College of Tianjin Medical University, Tianjin, China; ^3^ Department of Graduate School, Sun Yat-sen University, Sun Yat-Sen Memorial Hospital, Guangzhou, China; ^4^ Department of Graduate School of Tianjin Medical University, Tianjin, China; ^5^ Department of Clinical College of Neurology, Neurosurgery and Neurorehabilitation, Tianjin Medical University, Tianjin, China; ^6^ Department of Orthopaedic Surgery, Xiangyang Central Hospital, Affiliated Hospital of Hubei University of Arts and Science, Xiangyang, China

**Keywords:** cancer, photodynamic therapy, bibliometrics, citespace, VOSviewer

## Abstract

A growing body of research has illuminated that photodynamic therapy (PDT) serves as an important therapeutic strategy in oncology and has become a hot topic in recent years. Although numerous papers related to cancer PDT (CPDT) have been published, no bibliometric studies have been conducted to summarize the research landscape, and highlight the research trends and hotspots in this field. This study collected 5,804 records on CPDT published between 2000 and 2021 from Web of Science Core Collection. Bibliometric analysis and visualization were conducted using VOSviewer, CiteSpace, and one online platform. The annual publication and citation results revealed significant increasing trends over the past 22 years. China and the United States, contributing 56.24% of the total publications, were the main driving force in this field. Chinese Academy of Sciences was the most prolific institution. *Photodiagnosis and Photodynamic Therapy* and *Photochemistry and Photobiology* were the most productive and most co-cited journals, respectively. All keywords were categorized into four clusters including studies on nanomaterial technology, clinical applications, mechanism, and photosensitizers. “nanotech-based PDT” and “enhanced PDT” were current research hotspots. In addition to several nano-related topics such as “nanosphere,” “nanoparticle,” “nanomaterial,” “nanoplatform,” “nanomedicine” and “gold nanoparticle,” the following topics including “photothermal therapy,” “metal organic framework,” “checkpoint blockade,” “tumor microenvironment,” “prodrug” also deserve further attention in the near future.

## Introduction

Cancer is undeniably one of the most complex and life-threatening diseases with rising morbidity and extremely high mortality rate, posing a tremendous threat to human health as well as a substantial economic burden on the whole society (Bray et al., 2018; Sung et al., 2021). Conventional cancer treatment options include surgical intervention, chemotherapy, and radiotherapy. However, the results of these treatments are generally unsatisfactory and often associated with significant side effects. At the moment, the search for more effective alternative therapies with minimal off-target side effects is of urgent need (Kaplan et al., 2013). Photodynamic therapy (PDT), a non-invasive cancer therapeutic modality with clinical appeal, has received increasing attention for their advantages such as high selectivity, minimally invasive nature, and low immunogenicity (Agostinis et al., 2011; Aniogo et al., 2021). To be effective, there are three necessary nontoxic components involved in the PDT process: a specific light source, the light-activated photosensitizer (PS) and molecular oxygen (Kwiatkowski et al., 2018). Briefly, cancer PDT (CPDT) is based on the ability of PS to specifically accumulate in tumor tissue and subsequent stimulate the production of reactive oxygen species (ROS) including singlet oxygen (^1^O2), hydroxyl radicals (·OH) and superoxide radicals (·O2^−^) under local exposure of irradiation with visible light of specific wavelength (Alvi et al., 2021; Hu et al., 2021). The resultant ROS can cause irreversible oxidative damage to tumor cells, induce microvascular injury, stimulate immune responses, and trigger a series of cell death mechanisms (Juarranz et al., 2008; Robertson et al., 2009). Elucidation of PDT mediated cell death pathways has been a long-standing effort in the field, and over years of investigation, extensive studies have confirmed that apoptosis, necrosis, autophagy, and paraptosis all played important roles in the field.

As of date, cancer ablative potential of PDT has been applied clinically to treat multiple types of cancers including skin cancer (Braathen et al., 2007; Zhang et al., 2022), head and neck cancer (Ahn et al., 2014; Meulemans et al., 2019), gastric cancer (Yano and Wang, 2020), brain cancer (Li et al., 2022), breast cancer (George and Abrahamse, 2016; Dos Santos et al., 2020), non-small-cell lung cancer (Chhatre et al., 2021), genitourinary cancer (Bozzini et al., 2012; Osuchowski et al., 2021), and more, and most of which achieved satisfactory clinical effect. In spite of this, the depth and breadth of its clinical application have not been fully exploited due to the limitations such as oxygen deficiency within the tumor microenvironment, inadequate light penetration depth, insufficient bioavailability and targeting of PSs, and the immune escape of tumor cells (O'Connor et al., 2009; Cheng et al., 2015). Motivated by the aforementioned limitations, numerous investigations put their emphasis on increasing the selectivity of PSs accumulation in tumor tissue and improve its tumor targeting (Chen et al., 2015; Monro et al., 2019). Many studies have also been performed in regard to the development of light sources with stronger penetrability (Zhang et al., 2020). As a result, numerous scientific literature on CPDT has been published over the past several years.

Bibliometrics is a tool that integrates mathematical and statistical approaches to qualitatively and quantitatively evaluate academic literature in a specific field (Shen et al., 2022). It provides an opportunity to analyze the development trend in a particular field from a global perspective, and also investigate the contribution of individuals from different institutions and countries. At present, as a burgeoning method, it has been widely applied on numerous medical fields (Wu et al., 2021a; Wu et al., 2021b). Take the field of oncology as an example, numerous previous studies have analyzed the publication trend and research hotspots of various types of tumors though bibliometric approaches (Ahn and Hwang, 2020; Gu et al., 2021; Shi et al., 2021). In recent years, along with the development of various interventional techniques for malignant tumors such as chimeric antigen receptor (CAR)-based immunotherapy (Pouliliou et al., 2020; Ou et al., 2022), programmed cell death 1 (PD-1)/programmed cell death ligand 1 (PD-L1) inhibitors treatment (Sun et al., 2021; Liu et al., 2021); molecular targeted therapy (Sheahan et al., 2018), monoclonal antibodies (Kho and Brouwers, 2012), *etc*. many scholars have also conducted bibliometric studies to make a comprehensive analysis of these novel therapeutic strategies. Whereas, to the best of our knowledge, no bibliometric studies have yet been conducted to evaluate the global development trend of CPDT. To fill this gap, we searched the studies on CPDT published during 2000–2021. This bibliometric study was conducted to address the following questions:

Q1: What is the global development trend on the field of CPDT based on the information from published literature?
Q2: What are the most prolific and influential countries/regions, institutions, authors in this field?
Q3: What are the most preferred journals for publishing documents related to CPDT?
Q4: What are the main research directions and hotspots? How they changed over time?
Q5: What are the most concerned research frontiers and potential hotspots in the near future?

## Materials and Methods

### Data Sources and Search Strategies

**FIGURE 1:**
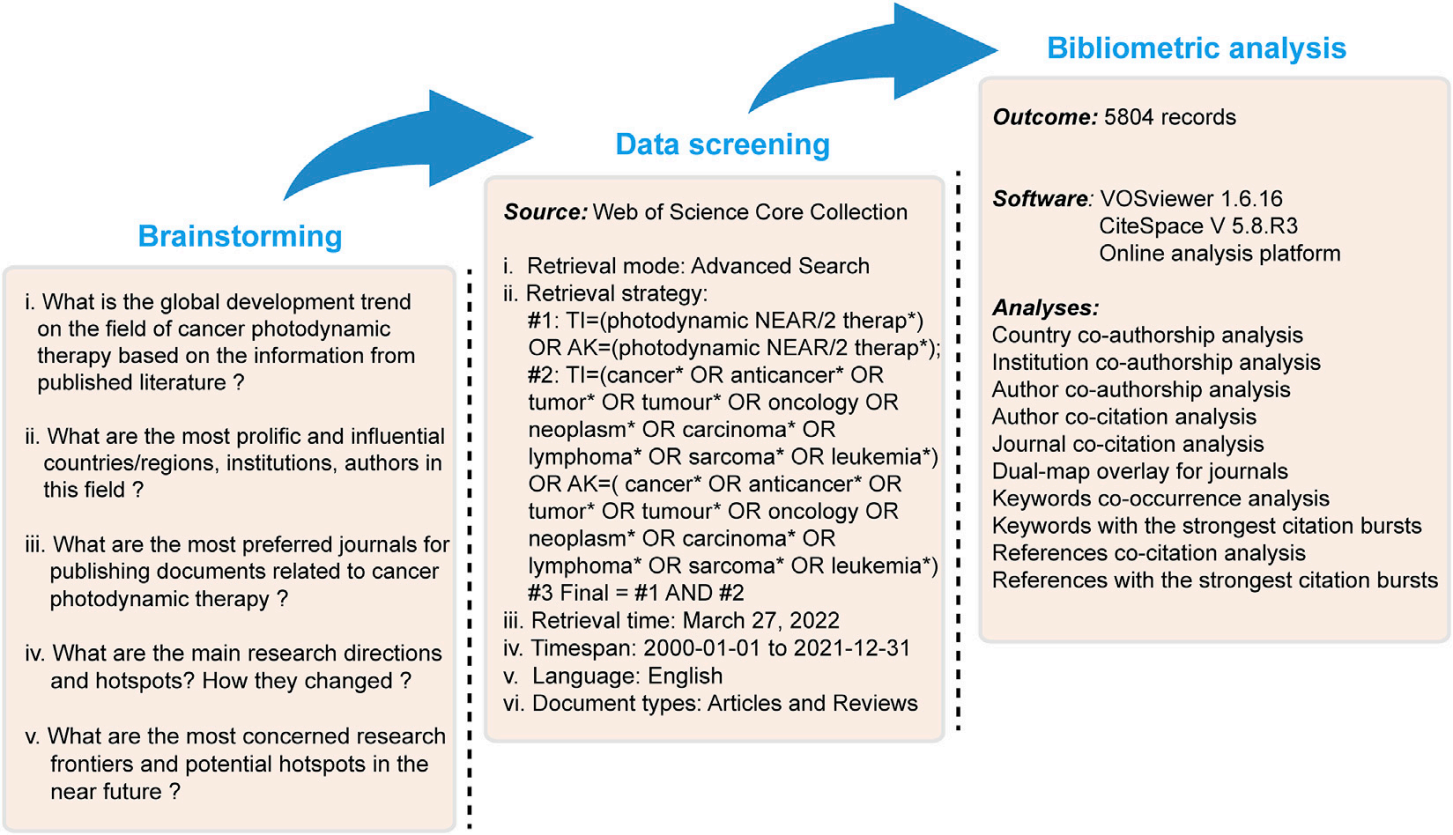
Conceptual design and flowchart of the study.

Given the consideration of the quality of the eligible literature, as well as the requirement for appropriate reference format, we adopted the Science Citation Index-Expanded of Web of Science Core Collection (WoSCC) as the data source. The WoSCC, covering more than 12,000 of high-quality scientific journals, is known to harbor relatively reliable database and also considered as the optimal database in previous bibliometric studies (Yeung, 2019a; Yeung et al., 2020; Wu et al., 2021c; Ma et al., 2022). All searches were carried out independently by two investigators (CK and GQ) on a single day, 27 March 2022, to avoid the significant bias arising from rapid database renewal. The search strategy was as follows: #1: TI=(photodynamic NEAR/2 therap*) OR AK=(photodynamic NEAR/2 therap*); #2: TI=(cancer* OR anticancer* OR tumor* OR tumour* OR oncology OR neoplasm* OR carcinoma* OR lymphoma* OR sarcoma* OR leukemia*) OR AK=(cancer* OR anticancer* OR tumor* OR tumour* OR oncology OR neoplasm* OR carcinoma* OR lymphoma* OR sarcoma* OR leukemia*); Final dataset: #1 AND #2. To capture as many data sources as possible, the wildcard character (*) that could be substituted for any other characters and allows variable endings of keywords was used. For example, “cancer*” would also return the terms of “cancer” and “cancers.” In addition, the wildcard character of NEAR/2 was used to search for two words, in an arbitrary order, separated by a maximum of two terms (e.g., photodynamic NEAR/2 therap* would have identified “photodynamic therapy” and “photodynamic cancer therapy”). The period of interest was from 2000 to 2021. The publication types were restricted to original articles and reviews and only English language literature was included. The conceptual design and flowchart of the study is presented in Figure 1.

### Data Extraction and Collection

By using the “Export Records to File” option of WoSCC, all results were downloaded with the record content of “Full Record and Cited References” and exported as plain text or tab-delimited format for additional processing. Bibliographic information including annual number of publications and citations, countries, institutions, authors, journals, funding agencies, research areas, keywords, and references was summarized and saved in a Microsoft Excel file. The impact factor (IF) and subject category quartile ranks of journals was derived from the 2020 Journal Citation Reports (JCR). The H-index is defined as the number of papers (h) that have received at least h citations, which is often used to measure the cumulative impact of a country’s output. That is to say, a country with an H-index of 20 means that 20 documents of which have at least 20 citations each. Qualitative and quantitative analyses were conducted by VOSviewer 1.6.16 (Leiden University, Netherlands), CiteSpace V 5.8.R3 (Drexel University, United States), and one online analysis platform (https://bibliometric.com).

### Bibliometric Analysis

First, the online bibliometric analysis platform was used to perform collaboration analysis of countries. The annual publication trend analysis of the productive countries was also carried out.

VOSviewer, developed by Van Eck and Waltman from Leiden University in 2009, is a widely utilized software tool for visualizing and constructing bibliometric network maps (van-Eck and Waltman, 2010). In the current study, it was utilized to visualize co-authorship analysis of countries/institutions/authors, co-citation analysis of journals, and co-occurrence analysis of keywords. “fractional counting” was the counting method and several VOSviewer thesaurus files were used to merge different variants. VOSviewer could generate three different visualization maps including the network, density or overlay visualization maps with different meanings. Collectively, in these knowledge maps, node on the map represents an element such as a country, institute, or author *etc*. Links between the nodes can represent the relationship between elements. The link lines between nodes indicate relationships, and many factors determine the size of an element including number of publications, and frequency of citations or occurrences. The nodes and lines were colored difference according to different clusters or corresponding average appearing year (AAY) (Yeung et al., 2019b).

CiteSpace software, developed by Chen Chaomei from Drexel University, is another Java-based bibliometric tool to analyze the development dynamics and research clusters in specific topics (Synnestvedt et al., 2005). We used CiteSpace to visualize institutional cooperation analysis, co-occurring subject categories, co-citation analysis of references and identified the references or keywords that received a good deal of attention in a certain time period, that is also called bursts detection. In addition, CiteSpace also has a unique dual-map analysis module, which could display knowledge flow, citation trajectories, and the topic distribution of academic journals (Ou et al., 2022). The parameters of CiteSpace were as follows: time span (2000/01/01 to 2021/12/31), year of slice (2), selection criteria g-index (k = 25), selection criteria (Top 50), link retaining factor (LRF = 3), look back years (LBY = 5), pruning (minimum spanning tree, pruning sliced networks).

### Statistical Analysis

R software (v3.6.3.), Microsoft Excel 2019, and SPSS (IBM SPSS Statistics 21, Inc., Chicago, IL, United States) were used for descriptive statistical analysis and plotting graphs. Curve fitting of the annual number of publications and citations was performed using Microsoft Excel. The best fitting model was selected based on the highest correlation coefficient (*R* ^*2*^). We calculated the growth rate of publications according to the specific calculation formula described by Wu et al. (Wu et al., 2021a). To test correlation between publications and citations, Pearson’s correlation coefficient was calculated and correlations were considered significant with a *p* value less than 0.05.

## Results

### Analysis of Annual Publication and Citation Trends

**FIGURE 2:**
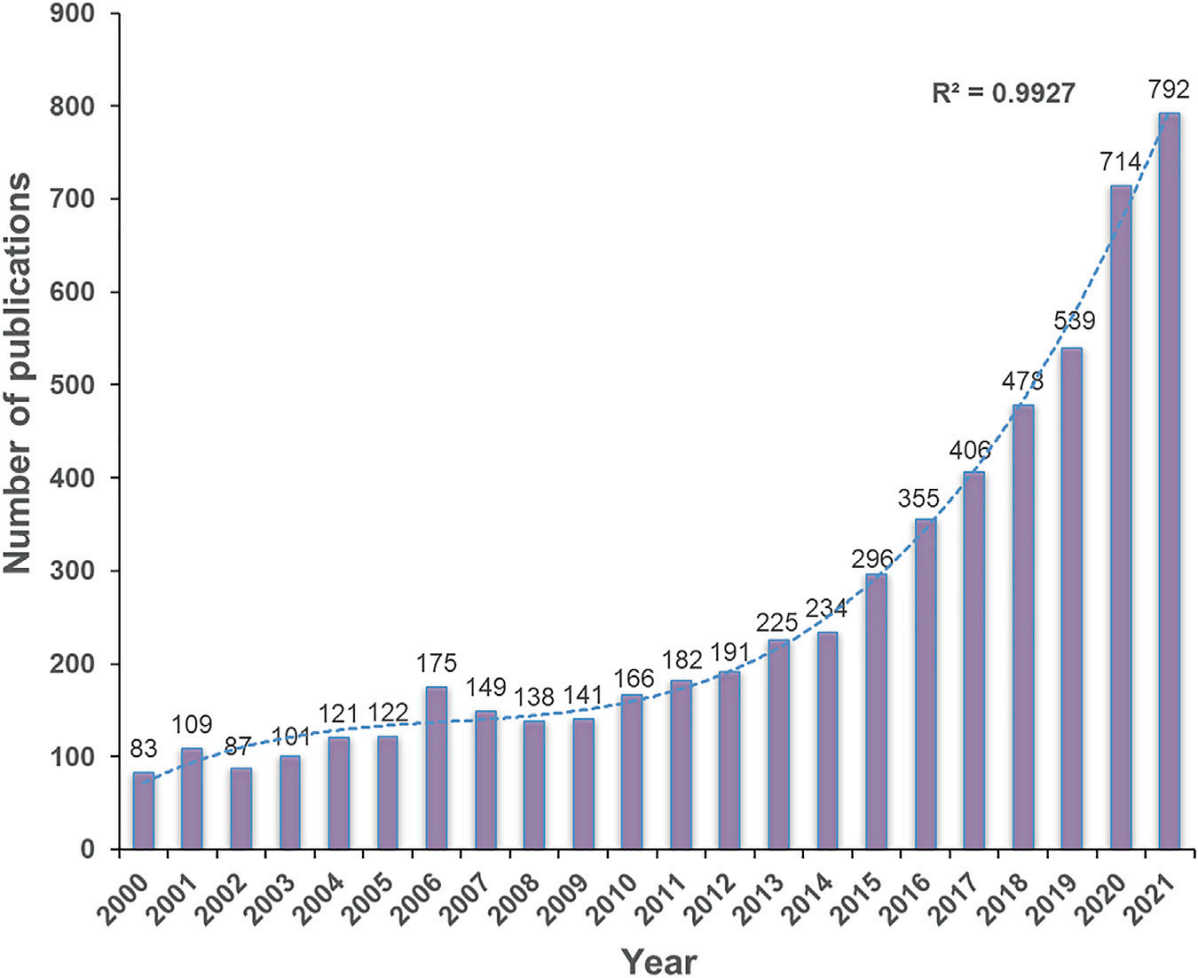
The annual publication trends in the past 22 years. Purple bars represent the number of papers related to CPDT per year. The blue dotted line represents the trend-fitted curve and the correlation coefficients (*R* ^2^) is displayed in the figure.

A total of 5,804 publications on CPDT were published on the WoSCC from 1 January 2000 to 31 October 2021, including 5,017 (86.44%) articles and 787 reviews (13.56%). The total number of citations for the retrieved papers was 203,989 (159,400 times cited without self-citations), and the average citations per document was 35.15. The H-index of all the selected publications related to CPDT was 171. The annual number of publications from 2000 to 2021 was shown in Figure 2. Publication concerning CPDT has increased from 83 in 2000 to 792 in 2021, and the average growth rate was 11.3%. Model fitting curve revealed a significant upward trend over the past 22 years (*R* ^2^ = 0.9927). Correspondingly, the annual number of citations exhibited a similar growth trend, steadily increasing from 25 in 2000 to 40,826 in 2021, with a significant correlation coefficient (*R* ^2^ = 0.9989) (Supplementary Figure S1). The result of the correlation analysis between publications and citations was reported in Supplementary Figure S2, and a statistically significant correlation was observed (Pearson’s correlation coefficient 0.989, *p* < 0.001, *R* ^2^ linear 0.979).

### Contribution of Countries/Regions and Funding Agencies

**FIGURE 3:**
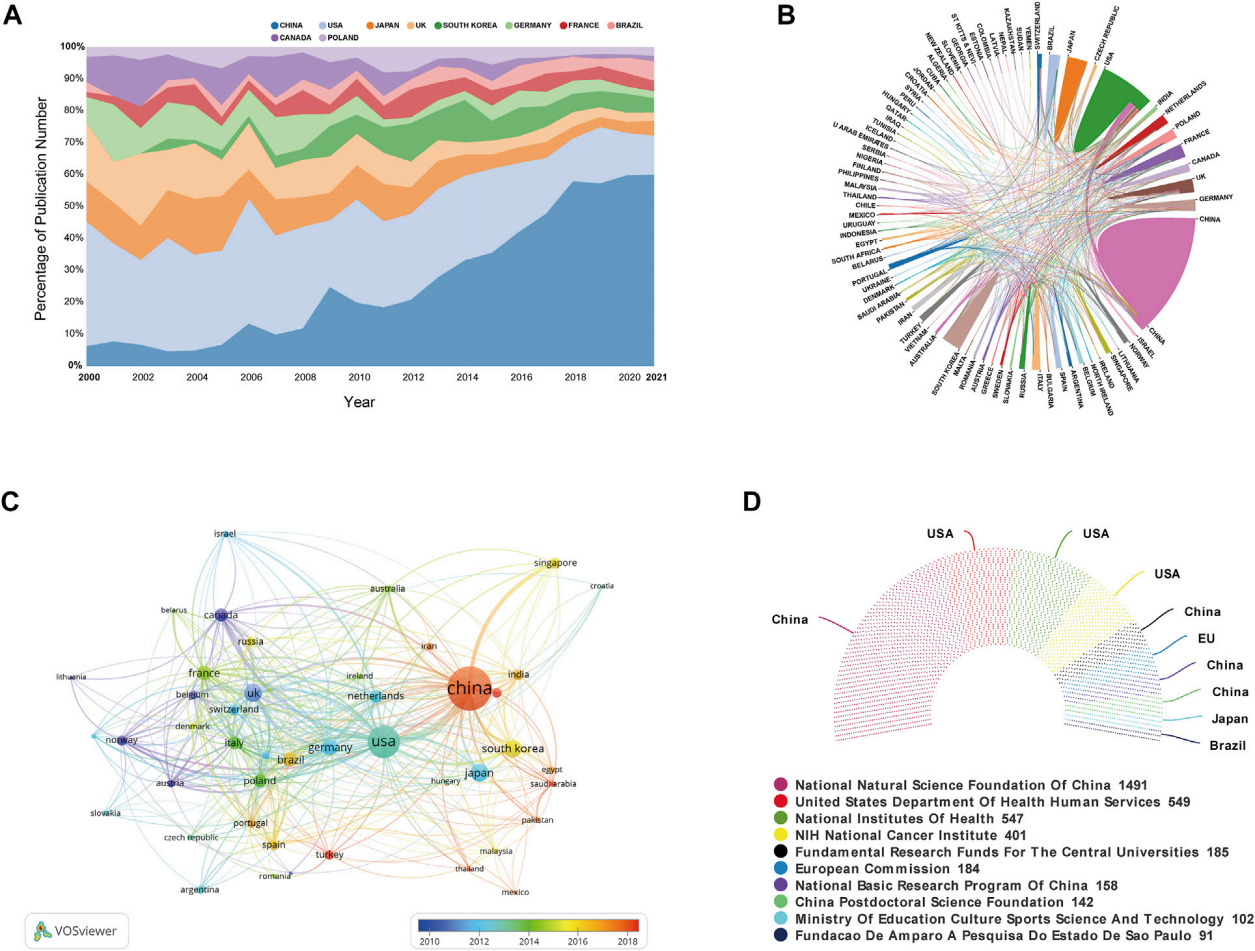
**(A)** The top 10 country’s change trend in the relative proportion of annual publications from 2000 to 2021. **(B)** Cooperation between contributed countries. Line thickness correlates with the intensity of the closeness. **(C)** Country co-authorship analysis by VOSviewer. In this overlay visualization map, each node is a country, and links between countries represent co-authorship relationship. The size of each node is proportional to the total number of publications. The node color reflects the corresponding average appearing year (AAY) according to the color gradient in the lower right corner. **(D)** The top 10 most active funding agencies involved in this domain.

All publications covered 84 countries/regions. China had published the most publications with 2,145 (36.96%) documents, followed by the United States [1,119 (19.28%)], Japan [341 (5.88%)], UK [332 (5.72%)], and South Korea [316 (5.44%)] (Table 1). China and the United States have contributed 56.24% of the total publications, far more than other countries. The top 10 country’s change trend in the relative proportion of annual publications from 2000 to 2021 was laid out in Figure 3A. China initially had trailed the United States in annual number of publications, but after 2008 its publications have increased rapidly and surpassed the United States in 2012, and still maintain a high-speed growth rate in 2021. Among the top 20 countries/regions according to publications, China has the highest H-index of 128, followed by the United States and UK. However, the mean number of citations in China was lower than other countries such as Norway, Canada, Singapore and the United States.

**TABLE 1:** Top 20 productive countries/regions related to CPDT.

Ranking	Countries	Publications, *n*	% Of 5,804	H-Index	Average citations per document
1	CHINA	2,145	36.96	128	35.58
2	USA	1,119	19.28	107	52.44
3	JAPAN	341	5.88	48	28.33
4	UK	332	5.72	65	49.02
5	SOUTH KOREA	316	5.44	46	31.17
6	GERMANY	246	4.24	52	37.88
7	FRANCE	221	3.81	46	34.76
8	BRAZIL	209	3.60	34	21.37
9	CANADA	184	3.17	48	61.51
10	POLAND	178	3.07	38	50.58
11	ITALY	168	2.89	40	29.4
12	NETHERLANDS	158	2.72	46	46.3
13	SINGAPORE	116	2.00	44	56.06
14	INDIA	114	1.96	26	20.08
15	SWITZERLAND	112	1.93	38	45.76
16	SPAIN	100	1.72	28	31.88
17	NORWAY	98	1.69	36	73.27
18	SOUTH AFRICA	88	1.52	21	21.6
19	RUSSIA	86	1.48	19	15.58
20	TURKEY	85	1.46	19	15.65

Ranking: according to the number of total publications.

The international collaboration network among countries (Figure 3B) showed that China mainly worked in close cooperation with the United States and Singapore. As shown in Figure 3C, country co-authorship was also conducted analysis by VOSviewer. A total of 47 countries/regions with the minimum number of 10 publications were included. As can be seen, China and the United States were situated in a central position of this overlay visualization map. According to the color gradient in the lower right corner, these countries such as China, Turkey, Saudi Arabia, *etc*. were marked with a red color. And corresponding to this, Norway, Canada, and Beigium, *etc*. were given a blue color. Moreover, we also summarized the top 10 most active funding agencies involved in this domain (Figure 3D). More than half of funding sources were from China and the United States.

### Contribution of Institutions

**FIGURE 4:**
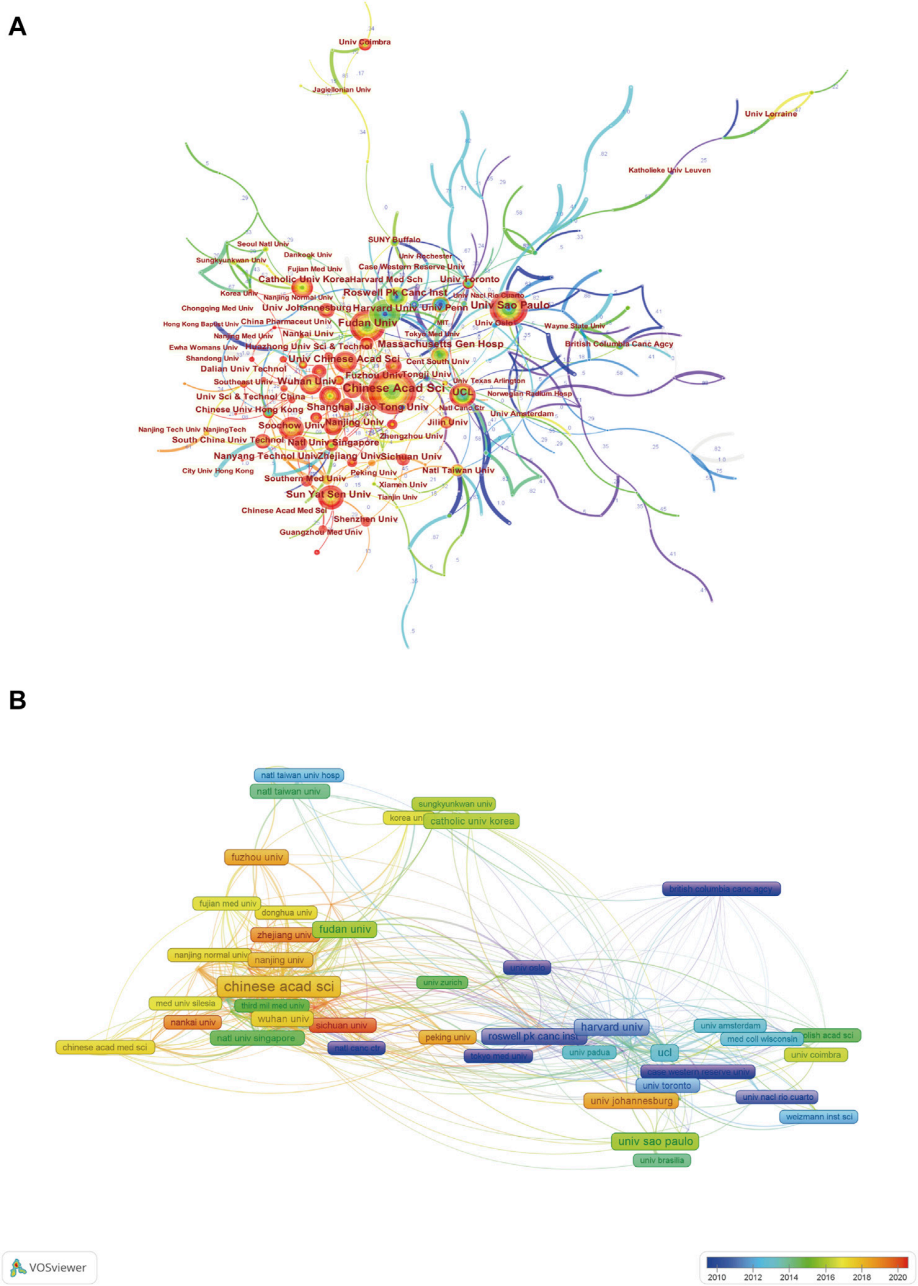
**(A)** Institutional collaboration analysis by CiteSpace. **(B)** Institution co-authorship analysis by VOSviewer.

Institutional collaboration analysis was performed by CiteSpace (Figure 4A). The size of the nodes grows proportionally to the number of publications. Four institutions with the highest number of publications were Chinese Academy of Sciences, Universidade de São Paulo, Fudan University, and University College London. As can be observed in the institution co-authorship network map generated by VOSviewer in Figure 4B. Only 114 institutions with a minimum of 20 publications were included. It is not difficult to see that international collaboration between institutions from different countries was not close enough and most of them conducted among domestic institutions. Additionally, the majority of institutions from China were given a red or yellow color with the larger AAY values.

### Contribution of Authors

**FIGURE 5:**
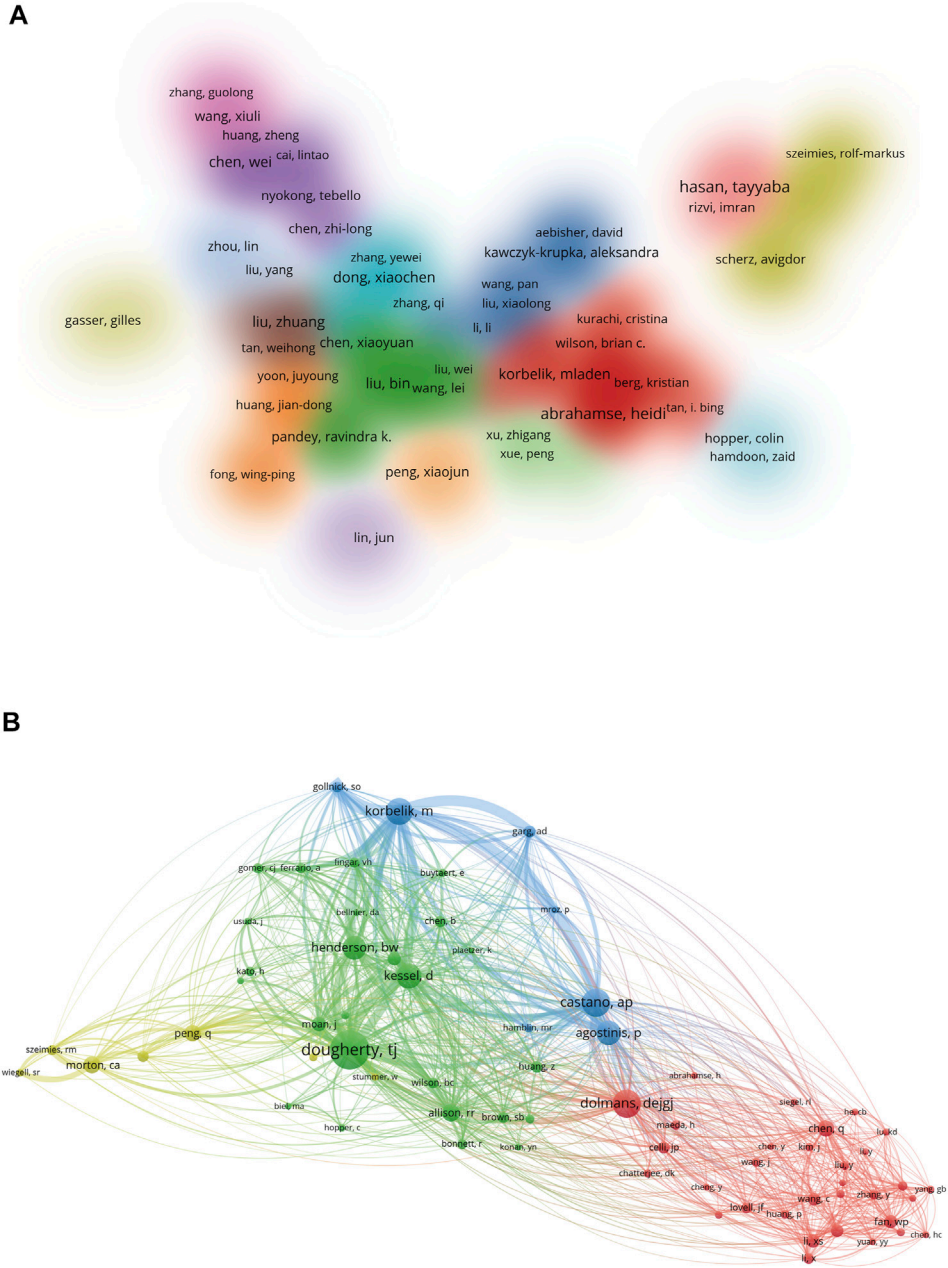
**(A)** Author co-authorship analysis by VOSviewer. In this cluster density map, authors with close relationship are allocated to one cluster with the same color. **(B)** Author co-citation analysis by VOSviewer. In this network visualization map, each node represents an author, and the lines connecting the nodes represent the co-citation relationship.

A cluster density map of author co-authorship analysis was generated by VOSviewer (Figure 5A). Of the included 214 authors (more than 10 papers), Hasan Tayyaba from Massachusetts General Hospital and Harvard Medical School contributed the highest number of publications, followed by Abrahamse Heidi from the University of Johannesburg, Zhang Xianzheng from Wuhan University. In addition, authors with close cooperative relationship in this map were assigned to one cluster with the same color, and a total of 16 author clusters were formed. Among them, author clusters from China occupied the most. In terms of author co-citation analysis, 71 authors with a minimum of 200 citations were included (Figure 5B). The top three authors with the largest total link strength (TLS) were Dougherty TJ, Korbelik M, and Castano AP.

### Journals and Co-Cited Journals

**FIGURE 6:**
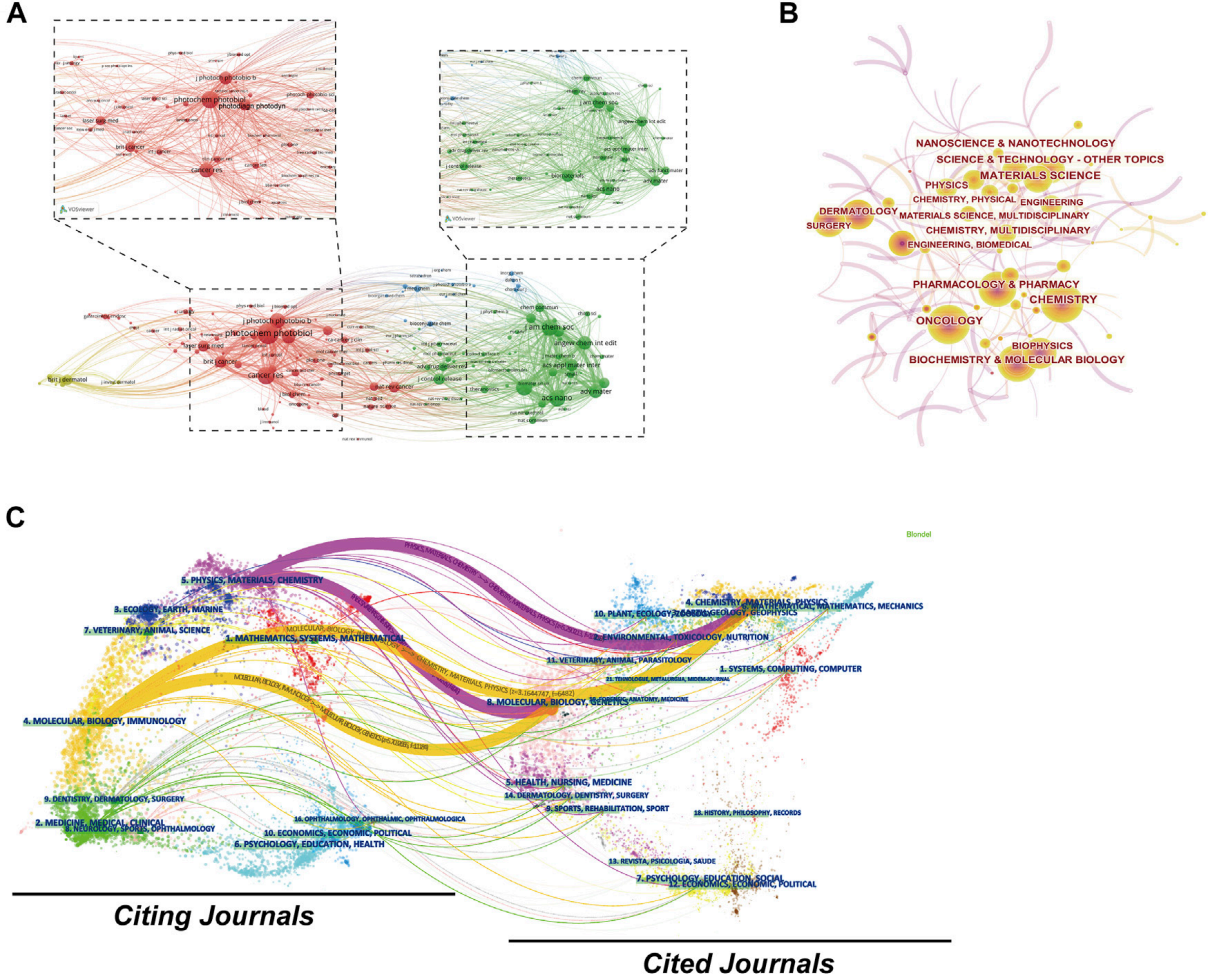
**(A)** Journal co-citation analysis by VOSviewer. **(B)** Co-occurring subject categories network of CPDT by CiteSpace. **(C)** The dual-map overlay of journals related to CPDT. In the dual-map, the citing journals are located on the left, and the cited journal is on the right. Colored paths indicate the citation relationships, with the thicker lines representing main pathways.

The top 20 most prolific journals were listed in Table 2. *Photodiagnosis and Photodynamic Therapy* (444 documents, 7.65%) published the most papers in this field, followed by *ACS Applied Materials Interfaces* (172 documents, 2.96%), *Journal of Photochemistry and Photobiology B Biology* (170 documents, 2.93%), *Biomaterials* (131 documents, 2.26%), and *Lasers in Surgery and Medicine* (116 documents, 2.00%). Among the top 20 most prolific journals, *Advanced Functional Materials* had the highest IF of 18.808, and 80% were classified as JCR Q1 or Q2. As indicated in Figure 6A, a co-citation map of journals was generated by VOSviewer. The minimum amounts of citations were set as 300. There were 162 journals that met the threshold. The most frequently co-cited journal was *Photochemistry and Photobiology*, with the TLS of 7,912.01, followed by *Cancer Research*, *ACS Nano*, *Biomaterials*, and *Journal of the American Chemical Society*. Figure 6B illustrated the visualization map of co-occurring subject categories. Top three subject categories ranked by counts were chemistry, materials science, and oncology. In addition, a dual-map overlay of journals was created to reflect the subject distribution of academic journals (Figure 6C). There were four colored primary citation pathways in this map.

**TABLE 2:** Top 20 most active journals in CPDT field.

Ranking	Sources title	Output	% Of 5,804	IF 2020	JCR quartile 2020
1	PHOTODIAGNOSIS AND PHOTODYNAMIC THERAPY	444	7.65	3.631	Q3
2	ACS APPLIED MATERIALS INTERFACES	172	2.96	9.229	Q1/Q1
3	JOURNAL OF PHOTOCHEMISTRY AND PHOTOBIOLOGY B BIOLOGY	170	2.93	6.252	Q1/Q1
4	BIOMATERIALS	131	2.26	12.479	Q1/Q1
5	LASERS IN SURGERY AND MEDICINE	116	2.00	4.025	Q1/Q1
6	ACS NANO	89	1.53	15.881	Q1/Q1/Q1/Q1
7	ADVANCED FUNCTIONAL MATERIALS	76	1.31	18.808	Q1/Q1/Q1/Q1/Q1/Q1
8	PHOTOCHEMISTRY AND PHOTOBIOLOGY	76	1.31	3.421	Q3/Q2
9	JOURNAL OF CONTROLLED RELEASE	67	1.15	9.776	Q1/Q1
10	THERANOSTICS	67	1.15	11.556	Q1/Q1
11	INTERNATIONAL JOURNAL OF MOLECULAR SCIENCES	63	1.09	5.924	Q1/Q2
12	LASERS IN MEDICAL SCIENCE	62	1.07	3.161	Q3/Q2
13	ANGEWANDTE CHEMIE INTERNATIONAL EDITION	60	1.03	15.336	Q1
14	CANCERS	59	1.02	6.639	Q1
15	PHOTOCHEMICAL PHOTOBIOLOGICAL SCIENCES	59	1.02	3.982	Q2/Q2/Q2
16	INTERNATIONAL JOURNAL OF NANOMEDICINE	58	1.00	6.4	Q2/Q1
17	JOURNAL OF PORPHYRINS AND PHTHALOCYANINES	57	0.98	1.811	Q3
18	JOURNAL OF BIOMEDICAL OPTICS	52	0.90	3.17	Q2/Q2/Q2
19	NANOSCALE	51	0.88	7.79	Q1/Q1/Q2/Q1
20	MOLECULES	49	0.84	4.412	Q2/Q2

Ranking: according to the number of total publications.

### Keywords Co-Occurrence Analysis

**FIGURE 7:**
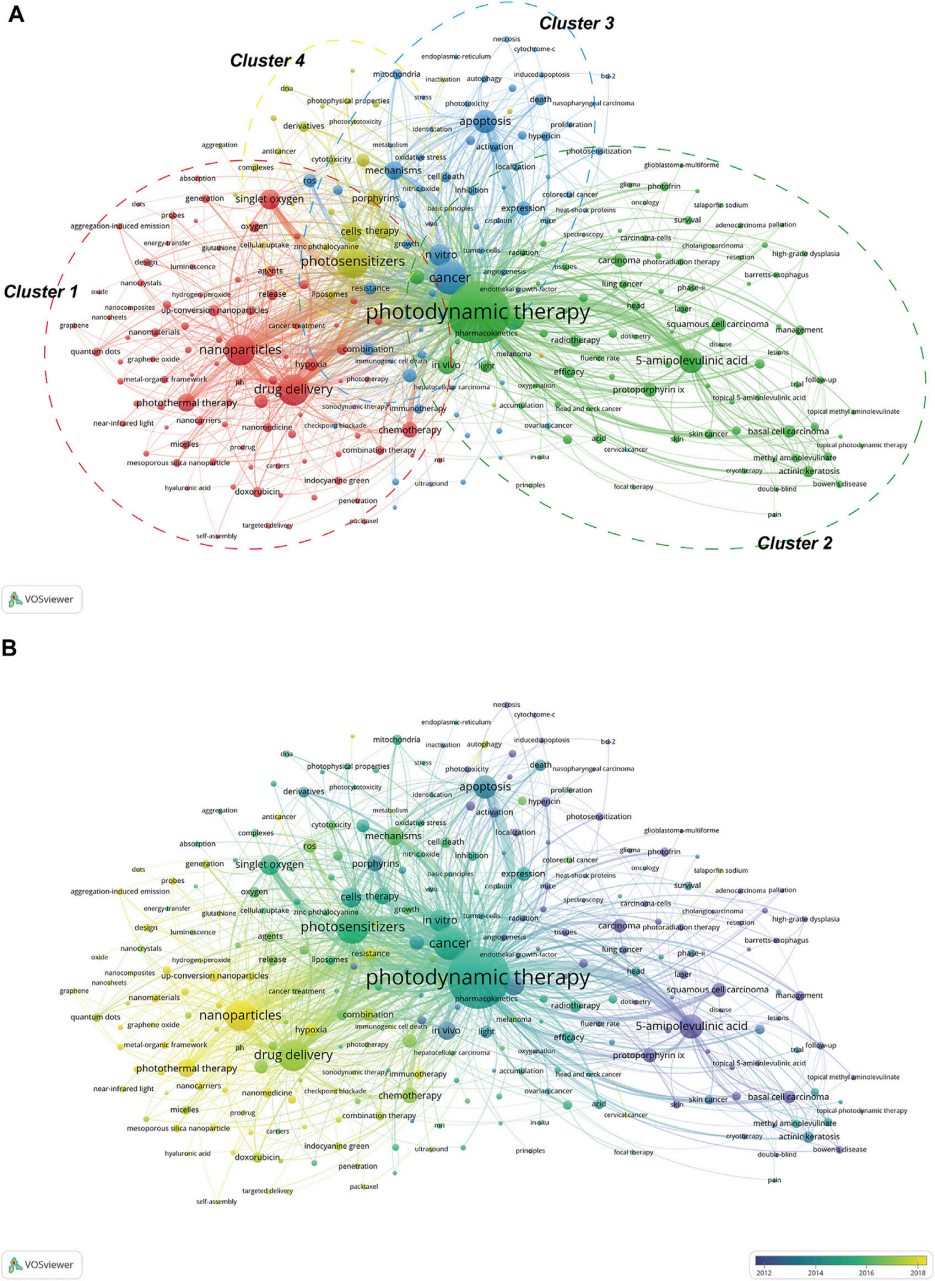
**(A)** Network visualization map of keywords co-occurrence analysis. In this network map, keywords with close relationship are assigned to one cluster with the same color. All the keywords could be divided into four clusters: cluster 1 (red nodes), cluster 2 (green nodes), cluster 3 (blue nodes), and cluster 4 (yellow nodes). **(B)** overlay visualization map of keywords co-occurrence analysis. The node color reflects the corresponding AAY according to the color gradient in the lower right corner. The nodes marked with purple or blue color represent the keywords that appeared relatively earlier, whereas keywords coded with yellow color represents the current research focuses.

Keywords are the essence of academic papers, and thus keywords analysis is an essential indicator of research hotspot. In this study, the keywords co-occurrence network map was constructed with VOSviewer. After manually merging several keywords with the same meaning and deleting meaningless keywords, a total of 276 keywords with at least 30 occurrence times were extracted from the 5,804 publications. Among them, “photodynamic therapy,” “cancer,” “photosensitizers,” “drug delivery,” and “nanoparticles” were the top five keywords that appeared most frequently. Supplementary Table S1 summarized the information of the top 20 most frequent occurrences keywords. In addition, VOSviewer has a function to automatically divide all keywords into several major clusters. As shown in Figure 7A, all the keywords could be classified into four categories as follows: cluster 1 (red nodes, studies on nanomaterial technology), cluster 2 (green nodes, studies on the clinical applications), cluster 3 (blue nodes, studies on mechanism), and cluster 4 (yellow nodes, studies on photosensitizers). In addition to this, an overlay visualization map of keywords co-occurrence analysis was presented in Figure 7B. It is easy to observe that the current hot topics are mostly concentrated in cluster 1.

### Keywords Burst Analysis

**FIGURE 8:**
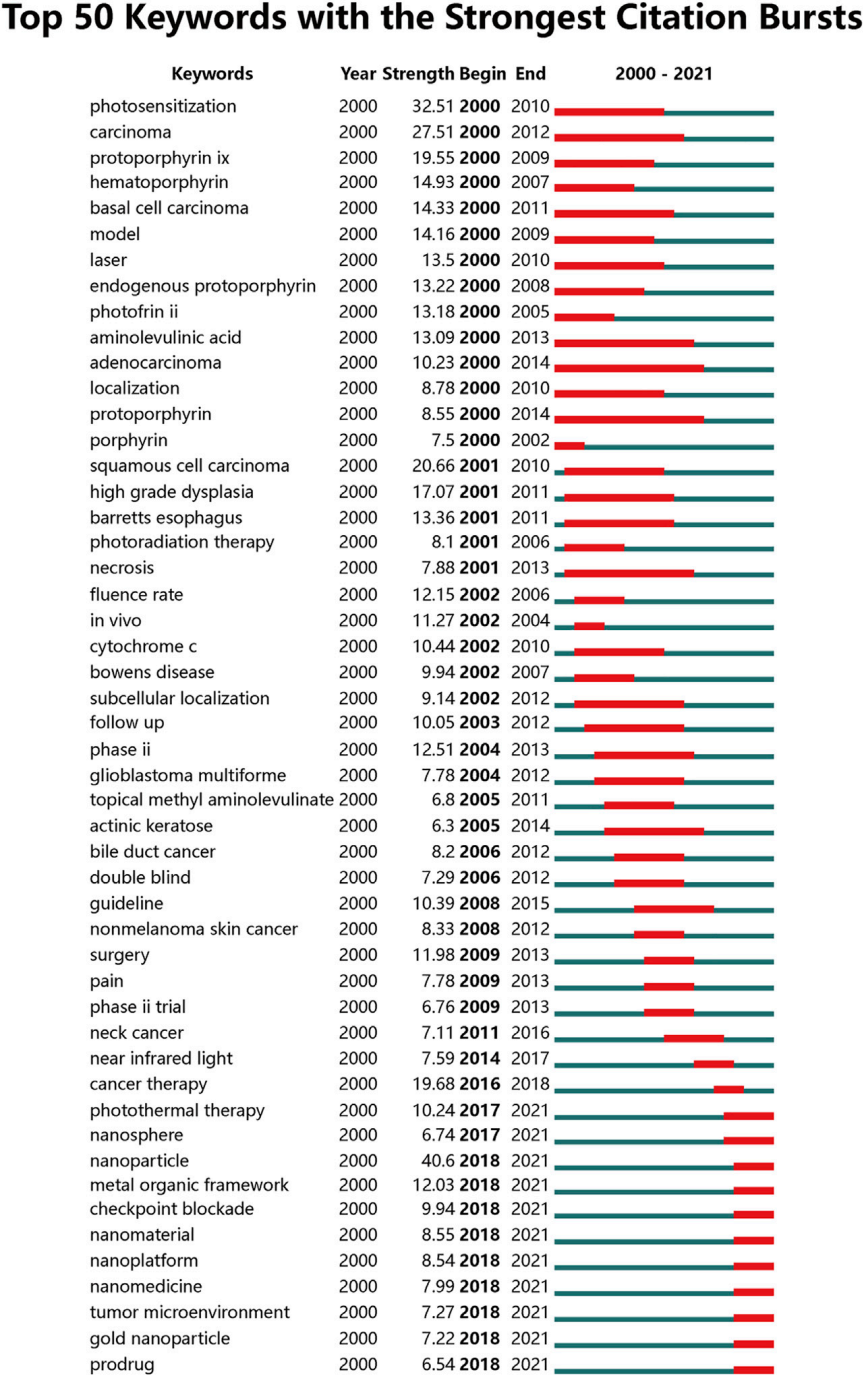
Top 50 keywords with the strongest citation bursts by CiteSpace. A blue line indicates the timeline, and the bars in red stands for a burst period including the beginning year, the end year, and the burst duration of the keywords.

CiteSpace was utilized to detect emergent keywords, and the top 50 keywords with the strongest citation bursts were presented in Figure 8. Among them, we mainly focused on these with ongoing bursts till 2021, including “photothermal therapy” (burst strength of 10.24), “nanosphere” (burst strength of 6.74), “nanoparticle” (burst strength of 40.6), “metal organic framework” (burst strength of 12.03), “checkpoint blockade” (burst strength of 9.94), “nanomaterial” (burst strength of 8.55), “nanoplatform” (burst strength of 8.54), “nanomedicine” (burst strength of 7.99), “tumor microenvironment” (burst strength of 7.27), “gold nanoparticle” (burst strength of 7.22), “prodrug” (burst strength of 6.54).

### References and Co-Cited References

**FIGURE 9:**
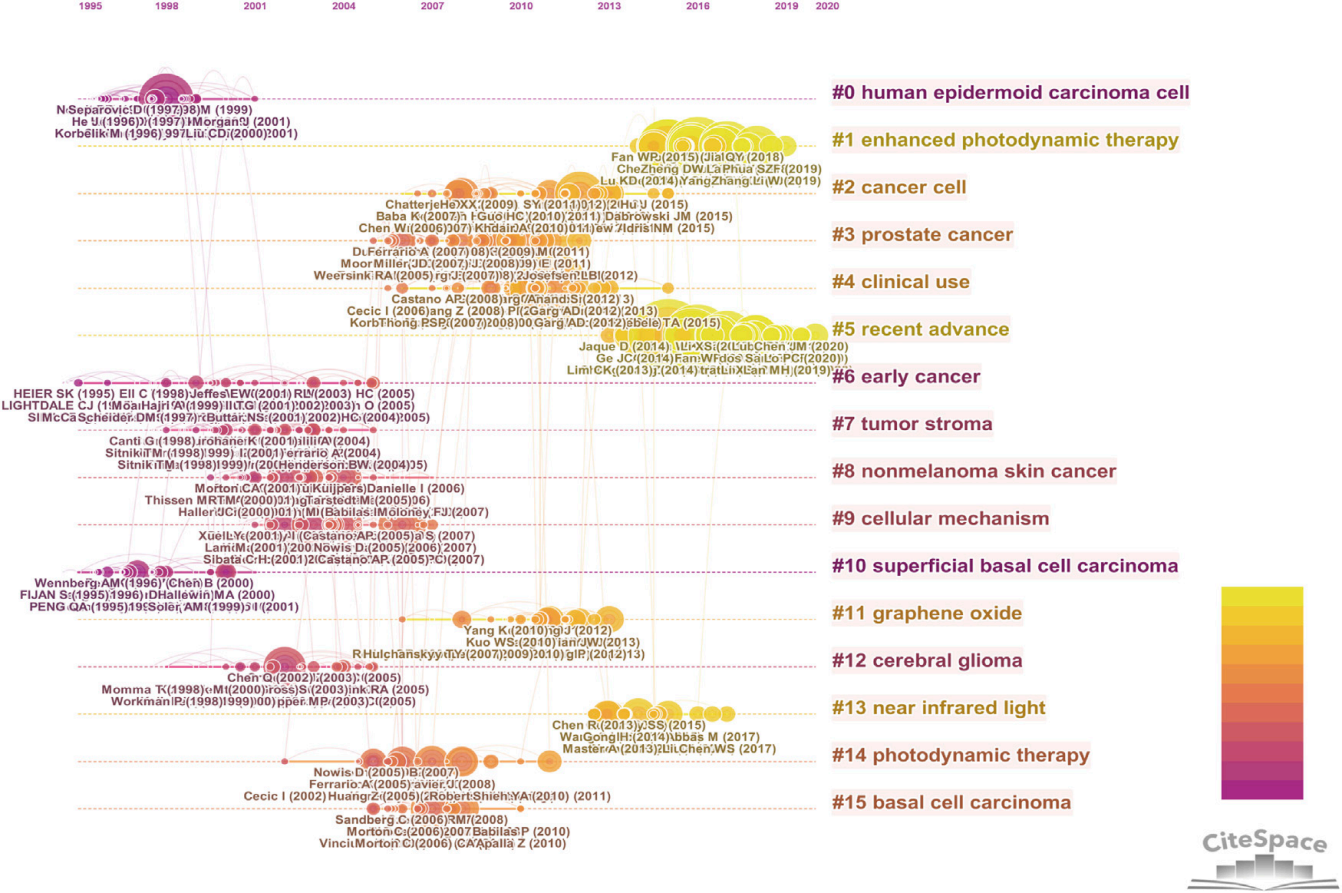
Reference co-citation analysis by CiteSpace. In this timeline view map, the position of the node on the horizontal axis indicates the time point of the first appearance, and lines connecting the nodes represent co-cited relationships. The node’s size is proportional to the number of citations of the reference. The more yellow the color means closer to 2021, while the redder the color means closer to 2000.

The top 20 most highly cited publications in CPDT field were shown in Table 3. Of them, the majority of the papers were reviews and all of which were cited more than 470 times. We have conducted reference co-citation analysis with CiteSpace. As shown in Figure 9 and Supplementary Table S2, all the included references were grouped into 16 clusters based on their major research topic. We found that “human epidermoid carcinoma cell” (Cluster 0) and “superficial basal cell carcinoma” (Cluster 10) and “early cancer” (Cluster 6) were relatively early hotspots, while “enhanced photodynamic therapy” (Cluster 1) is a focus of research attention currently. Besides that, references with strong citation bursts were also explored with CiteSpace (Supplementary Figure S3). References with citation bursts first appeared in 2000, and the most recent references with citation bursts appeared in 2016.

**TABLE 3:** Top 20 highly cited publications in CPDT field.

Ranking	Title	Total citations	Average citation per year	Journal	First Author	Published year
1	Photodynamic Therapy of Cancer: An Update	2,944	245.33	*CA-A CANCER JOURNAL FOR CLINICIANS*	Agostinis, Patrizia	2011
2	Photodynamic therapy and anti-tumour immunity	1,712	100.71	*NATURE REVIEWS CANCER*	Castano, Ana P	2006
3	The role of porphyrin chemistry in tumor imaging and photodynamic therapy	1,399	116.58	*CHEMICAL SOCIETY REVIEWS*	Ethirajan, Manivannan	2011
4	The present and future role of photodynamic therapy in cancer treatment	1,318	69.37	*LANCET ONCOLOGY*	Brown, SB	2004
5	Reactive oxygen species generating systems meeting challenges of photodynamic cancer therapy	944	134.86	*CHEMICAL SOCIETY REVIEWS*	Zhou, Zijian	2016
6	Porphyrin and Nonporphyrin Photosensitizers in Oncology: Preclinical and Clinical Advances in Photodynamic Therapy	840	60	*PHOTOCHEMISTRY AND PHOTOBIOLOGY*	O'Connor	2009
7	Current state, achievements, and future prospects of polymeric micelles as nanocarriers for drug and gene delivery	822	48.35	*PHARMACOLOGY AND THERAPEUTICS*	Nishiyama, Nobuhiro	2006
8	Photodynamic therapy (PDT): A short review on cellular mechanisms and cancer research applications for PDT	746	53.29	*JOURNAL OF PHOTOCHEMISTRY AND PHOTOBIOLOGY B-BIOLOGY*	Robertson, C. A	2009
9	Ceramic-based nanoparticles entrapping water-insoluble photosensitizing anticancer drugs: A novel drug-carrier system for photodynamic therapy	731	36.55	*JOURNAL OF THE AMERICAN CHEMICAL SOCIETY*	Roy, I	2003
10	Near-infrared light induced *in vivo* photodynamic therapy of cancer based on upconversion nanoparticles	640	53.33	*BIOMATERIALS*	Wang, Chao	2011
11	Highly efficient drug delivery with gold nanoparticle vectors for *in vivo* photodynamic therapy of cancer	586	39.07	*JOURNAL OF THE AMERICAN CHEMICAL SOCIETY*	Cheng, Yu	2008
12	H2O2-Activatable and O-2-Evolving Nanoparticles for Highly Efficient and Selective Photodynamic Therapy against Hypoxic Tumor Cells	563	70.38	*JOURNAL OF THE AMERICAN CHEMICAL SOCIETY*	Chen, Huachao	2015
13	Photodynamic therapy in oncology	526	30.94	*ONCOLOGIST*	Triesscheijn, Martijn	2006
14	Perfluorocarbon nanoparticles enhance reactive oxygen levels and tumour growth inhibition in photodynamic therapy	525	65.63	*NATURE COMMUNICATIONS*	Cheng, Yuhao	2015
15	Methylene blue in photodynamic therapy: From basic mechanisms to clinical applications	525	29.17	*PHOTODIAGNOSIS AND PHOTODYNAMIC THERAPY*	Tardivo, Joao Paulo	2005
16	Photodynamic therapy - mechanisms, photosensitizers and combinations	500	100	*BIOMEDICINE AND PHARMACOTHERAPY*	Kwiatkowski, Stanislaw	2018
17	Smart Human Serum Albumin-Indocyanine Green Nanoparticles Generated by Programmed Assembly for Dual-Modal Imaging-Guided Cancer Synergistic Phototherapy	498	55.33	*ACS NANO*	Sheng, Zonghai	2014
18	Guidelines on the use of photodynamic therapy for nonmelanoma skin cancer: An international consensus	493	30.81	*JOURNAL OF THE AMERICAN ACADEMY OF DERMATOLOGY*	Braathen, Lasse R	2007
19	Photodynamic therapy of cancer. Basic principles and applications	483	32.2	*CLINICAL AND TRANSLATIONAL ONCOLOGY*	Juarranz, Angeles	2008
20	Transition Metal Complexes and Photodynamic Therapy from a Tumor-Centered Approach: Challenges, Opportunities, and Highlights from the Development of TLD1433	472	118	*CHEMICAL REVIEWS*	Monro, Susan	2019

Ranking: according to the number of total citations.

## Discussion

### Answer for Q1: What Is the Global Development Trend on the Field of CPDT Based on the Information From Published Literature?

Generally speaking, annual publication and citation counts are the most intuitive indicator to reflect the research interest of scholars in a specified field (Pouliliou et al., 2020; Ou et al., 2022). As can be observed in the annual number of publications, it exhibited a significant upward trend over the past 22 years. We could roughly divide it into three periods, slow growth period (2000–2012), acceleration period (2013–2019) and rapid growth period (2020–2021). The annual number of publications experienced a relatively slow growth tendency before 2012, and the production did not exceed 200 papers per year. Although PDT has already been approved clinically for tumor treatment in the 1990s, the developments of this therapeutic procedure still evolved slowly limited by photosensitizers and light sources. Since 2013, with continuous breakthroughs in nanotechnology and laser techniques, the number of publications on CPDT begins to appear a first acceleration period, and passed the mark of 500 papers in 2019 (Cai and Sheng, 2015). In recent 2 years, the number continues to rise by a substantial margin, reaching more than 700 documents per year, and almost 25.9% of them (1,506 papers) were published over the last 2 years. Correspondingly, the annual number of citations exhibited a similar growth trend, and has a positive relationship with publications, suggesting that the variations in the citation change trend could be explained by the rapid growth of publication. From the results mentioned above, it is evident that research on CPDT has gained increasing attention recently. With the breakthroughs and advents in the field of clinical biomaterial and optical imaging, we believed that there will be bright prospects for CPDT research in the future.

### Answer for Q2: What Are the Most Prolific and Influential Countries/Regions, Institutions, Authors in This Field?

As can be seen from top 20 productive countries/regions related to CPDT in Table 1, most of them are concentrated in the continents of the Northern Hemisphere, such as East Asian, North American as well as many European countries. According to statistics, China ranked first in terms of the total number of publications and H-index values in the field of CPDT research, followed by the United States, and undoubtedly, China and the United States contributed the most. As for the possible reasons, that is inseparable from significant investment by governments (Feng and Pei, 2011; Dou, 2020). In the top 10 most active funding agencies involved in this domain, more than half of funding sources were from China and the United States. As for institutions, Chinese Academy of Sciences, Universidade de São Paulo, Fudan University, and University College London are the top four institutions published the most CPDT related documents. It is evident from Figures 4A,B, the fact that China has occupied the most productive institutions in this field could partially explain why China consistently maintains its high quantity of publications. And consistent with the result from country analysis, most of the Chinese institutions have a larger AAY value, suggesting most of the them were the new entrants in this field. In addition, it is worth noting that the international collaboration between institutions from different countries was not close enough and most of them conducted among domestic institutions.

In terms of authors, Hasan Tayyaba from Massachusetts General Hospital and Harvard Medical School, Abrahamse Heidi from the University of Johannesburg, and Zhang Xianzheng from Wuhan University contributed the highest number of papers on CPDT research. Hasan Tayyaba is mainly focused on the mechanistic, therapeutic, and imaging aspects of PDT to cancer (Rai et al., 2010; Hasan, 2012). And she and others also attempted to develop mechanism-based PDT combination strategies involving multiple inhibitors of oncogenic pathways for tumor therapy (Verma et al., 2007; Huang et al., 2016). While Abrahamse Heidi mainly explored various biocompatible nanoparticles and nanocarriers for enhanced CPDT applications (Dhilip Kumar and Abrahamse, 2021; Winifred Nompumelelo Simelane and Abrahamse, 2021). Similarly, Zhang Xianzheng focused on the investigation of nanoplatform or nanoparticles for enhanced PDT especially a variety of approaches to overcome hypoxic tumors (Zheng et al., 2016; Liu et al., 2017; Li et al., 2019). As for author co-citation analysis, the top three authors with the largest TLS were Dougherty TJ, Korbelik M, and Castano AP. Of note, although most of them have not published a large number of publications, they still occupied the core locations in the co-citation network map, suggesting that they have contributed high impact papers in this field (Roy et al., 2003; Agostinis et al., 2011). As expected, all of them are involved at least one highly cited paper in CPDT field. To sum up, these scholars were considered to play unique and indispensable roles in CPDT field either from a quantity or quality perspective. Their studies may continue to influence the development of the field and guide the cutting-edge research on CPDT in the future.

### Answer for Q3: What Are the Most Preferred Journals for Publishing Documents Related to CPDT?

Journal analysis is a common step of bibliometric analysis that could provide important information for researchers to choose the proper journals for submitting their studies. In previous bibliometric studies, journal analysis is usually an essential part (Ahn and Hwang, 2020; Wu et al., 2021a; Gu et al., 2021; Shi et al., 2021). Therefore, the characteristics of the most popular journals in this field were analysed. As can be seen from Table 2, *Photodiagnosis and Photodynamic Therapy*, *ACS Applied Materials Interfaces*, *Journal of Photochemistry and Photobiology B Biology*, *Biomaterials*, and *Lasers in Surgery and Medicine* were the top five most popular journals involved in the publication of research on CPDT. Consequently, it is reasonably concluded that future developments related to CPDT are more likely to be published in these journals. Additionally, in terms of the discipline classification of the top 20 journals, most of them belonging to Nanoscience and Nanotechnology, Chemistry, Materials Science, Biochemistry and Molecular Biology, and Oncology, which reflects the main research directions and focuses in this domain. Besides that, we also conducted the journal co-citation analysis to identify the core journals in this field. The journal co-citation analysis showed that *Photochemistry and Photobiology*, *Cancer Research*, *ACS Nano*, *Biomaterials*, and *Journal of the American Chemical Society* were the top five journals with the largest TLS. This result was mainly related to the publication of high-quality studies in these journals. Thus, hotspots and focus of future advances related to CPDT may also appear in these journals.

In addition, as can be seen from the dual-map overlay of journals, literature published to journals in the field of chemistry/materials/physics and molecular/biology/genetics were mainly cited by papers published in physics/materials/chemistry and molecular/biology/immunology. Although there also exist many links originating from medicine/medical/clinical, a primary citation pathway has not been formed yet. With further clinical trials widely being conducted in the future, it could be expected that these citation links from clinical use will increase at a rapid pace.

### Answer for Q4: What Are the Main Research Directions and Hotspots? How They Changed Over Time?

Generally, keywords and references analysis are the essence of academic papers, which could reflect the current research focus, help to understand the evolution trend, and predict the research prospect and hotspot in the future (Gu et al., 2021; Shi et al., 2021). In this part, we have used two kinds of bibliometric software to allow for comprehensive analysis. First, in the keywords co-occurrence network map, VOSviewer could assign keywords with close relationship to one cluster with the same color. As shown in Figure 7A, all of them could be classified into four categories. We could find that current research directions for CPDT mainly focus on studies on nanomaterial technology (Eisenbrey et al., 2018; Hu et al., 2019; Xavierselvan et al., 2021), clinical applications (Leroy et al., 2021; Yano et al., 2021), mechanism (Juarranz et al., 2008; Robertson et al., 2009), and photosensitizers (Zhang, et al., 2018). Besides that, in the overlay visualization map of keywords co-occurrence analysis, VOSviewer could impart keywords with different colors according to their AAY. The nodes marked with purple or blue color represent the keywords that appeared relatively earlier, whereas keywords coded with yellow color represents the current research focuses. As evident from Figure 7B, in the early stage, the CPDT research was mainly focused on “clinical applications” in cluster 2, whereas keywords belong to cluster 1 had the relatively larger AAY than other clusters, implying that the current research hotspot in the field has shifted to nanotech-based PDT. Already back in 1995, the U.S. Food and Drug Administration (FDA) has approved the first PS (Photofrin^®^) for the clinical treatment of esophageal cancer (Dolmans et al., 2003). As the research progresses, an increasing number of new PSs have been developed and approved. Of them, the second-generation PSs, such as 5-aminolevulinic acid (ALA), are currently attracting the most attention owing to high selectivity, low cytotoxicity, and rapid body clearance (Zhang, et al., 2018). Even so, there is still the lack of high-quality clinical studies on this subject due to the inherent drawbacks (O'Connor et al., 2009; Yano et al., 2021). In recent years, nanotech-based PDT is promising to solve this dilemma.

In addition to keywords, the time line view map of reference co-citation analysis by CiteSpace could also uncover the evolution process of a given field. The position of nodes on the horizontal axis indicates the time point of first appearance. The more yellow the color means closer to 2021, while the redder the color means closer to 2000. We could find that the earlier studies were mainly interested in “human epidermoid carcinoma cell” (Cluster 0) and “superficial basal cell carcinoma” (Cluster 10) and “early cancer” (Cluster 6), while the current stage cared more about “enhanced photodynamic therapy” (Cluster 1) and “recent advance” (Cluster 5).

As mentioned in the introduction, PDT, as an attractive and emerging therapeutic procedure, although has many advantages compared to chemoradiotherapy and surgical treatment. Its clinical application has not been fully exploited due to limitations such as less accumulation of agents, instability of PSs, and tumor hypoxia (O'Connor et al., 2009; Cheng et al., 2015). Currently, a growing number of investigations have focused on improving the efficacy of PDT by improving the stability of photosensitizing agents and penetrability of light sources (Wang et al., 2011), providing molecular oxygen directly or indirectly to tumor tissues (Eisenbrey et al., 2018; Sahu et al., 2020), as well as enhancing immune stimulation (Yang et al., 2022). In the meantime, several combined therapy strategies such as combining PDT with other antitumor agents (Brown et al., 2004; Biswas et al., 2015; George et al., 2017), photothermal therapy (Hou et al., 2020), sonodynamic therapy (Zheng et al., 2021), immunotherapy (Hua et al., 2021) and radiotherapy, could also improve the therapeutic effect of PDT.

Take tumor hypoxia as an example, hypoxic microenvironment is a common feature of various solid tumors, resulting in the ineffectiveness of the PDT process since the generation of toxic ROS requires the reaction of excited PS molecule with oxygen (Zhou et al., 2016). Thus, improvement of oxygenation in tumor tissues will not only alter the tumor microenvironment, but also improve the therapeutic efficacy of PDT and subsequent therapies. In an early phase, hypoxic regions of tumor tissues can be partially reversed with the use of hyperbaric oxygen (Tomaselli et al., 2001). Unfortunately, excess of oxygen may have harmful effects on multiple systems. In recent years, with spectacular advances in the development of nanomaterials and nanotechnology for targeted drug delivery, various oxygen-carrying nanoparticles and oxygen-generating nanomaterials have been developed for oxygen delivery inside the tumor. Among them, the oxygen-carrying nanosystems include oxygen-carrying nanobubbles and nanodroplets (Eisenbrey et al., 2018; Xavierselvan et al., 2021), perfluorocarbon-based O_2_ nanocarrier (Cheng et al., 2015; Hu et al., 2019), and hemoglobin-polymer conjugate as nanocarrier (Xu et al., 2018), and so on. Whereas the oxygen-generating nanosystems include MnO_2_ nanoparticles (Lin et al., 2018), nonfluorinated chitosan-chlorin e6/catalase nanoparticles (Chen et al., 2015; Sahu et al., 2020), biomimetic nanothylakoids (Ouyang et al., 2018), *etc*. Despite the fact that an increasing number of nanocarriers and nanoparticles have been discovered, this area of research is in its infancy and multitude of challenges faced in translational clinical applications. First, the design process of nanosystems at this stage is still complex with high safety risks, and unfavorable for large-scale clinical application. In addition, the long-term effectiveness, biocompatibility, biodegradability of nanomaterials are also very important issues deserving further consideration.

### Answer for Q5: What Are the Most Concerned Research Frontiers and Potential Hotspots in the Near Future?

By keywords co-occurrence analysis, we arrived at the conclusion that nanotech-based PDT was current research hotspots. In order to acquire more specific quantification metrics of each topic, CiteSpace was utilized to detect emergent keywords in a certain period. Burst detection is commonly used to detect keywords that have a surge of their appearance within a period of time, which is often considered an indicator of research frontiers over time (Ou et al., 2022). We have detected the top 50 keywords with the strongest citation bursts between 2000 and 2021 based on all the 5,804 publications. Among them, we mainly focused on those with ongoing bursts till 2021, suggesting that these topics have great potential to continue to be research frontiers in the future. It can be noted that the majority of keywords are still related to nanotechnology such as nanosphere, nanoparticle, nanomaterial, nanoplatform, nanomedicine, and gold nanoparticle. In addition to this, several other topics including photothermal therapy, metal organic framework, checkpoint blockade, tumor microenvironment, and prodrug are also worthy of attention. Of them, photothermal therapy is another phototherapeutic strategy mainly elevating the temperature in the tumor region to induce the death of targeted cells. In recent years, multiple studies have found that a combination of photothermal therapy and PDT could provide a synergetic effect for cancer treatment (Hou et al., 2020; Wang et al., 2020). Immune checkpoint blockade is an emerging cancer treatment method that has received great attention as a new milestone in the field. Previous studies have found that the release of immune substances during PDT could greatly improve the low immune response rate, and PDT induced antitumor immune response has higher therapeutic efficiency compare to monotherapy (Cramer et al., 2020; Zeng et al., 2022). Therefore, the combination of PDT and checkpoint blockade represents a highly promising strategy to cancer therapy. Nevertheless, the specific mechanism on the induction of the immune response by PDT needs further research. As for metal organic framework (MOF), it is a new class of porous materials and has been widely investigated as the anti-cancer drug delivery vehicle owing to its high drug-loading capacity, good biodegradability and easy functionalization (Feng et al., 2022). Several studies have found that PS-based MOFs could prevent self-aggregation of PS and significantly the PDT outcomes (Gao et al., 2020; Alves et al., 2021). MOFs with exceptionally high PS loading have emerged as the fourth-generation PS and achieved brilliant outcomes under pre-clinical studies. Undoubtedly, PS-based MOFs are currently emerging as a promising field with many contributions still to be made in the future.

### Limitations

Despite these interesting findings of this study, there are several limitations that need to be discussed. First, data on CPDT publications were only collected from the WoSCC database; thus, data from other relevant search engines such as PubMed, Embase, and Cochrane library were ignored. However, it is commonly accepted that WoSCC is the most suitable and recommended database for bibliometric analysis, and data from this database could depict most of the information of a certain field (Wu et al., 2021a; Shen et al., 2022). Few previous bibliometric studies have two or more databases due to the limitation of file formats (Braathen et al., 2007; Meulemans et al., 2019; Yano and Wang, 2020; Zhang et al., 2022). Second, it is difficult to accurately obtain all the related literature in this field since the diversity of keywords. We believe that the use of logical operators in the search strategy could greatly improve the accuracy of the retrieved results. Finally, several newly published and potentially high impact researches may not include in our analysis due to the low citation frequency. As a result, the scientific trends and hotspots of CPDT research may change with the bibliometric data updates.

## Conclusion

The present study is, to the best of our knowledge, the first to illustrate the current research status and global emerging trends in CPDT research using a bibliometric approach. Overall, the field of CPDT research is in a fast development phase and its area is likely to further expand in the future. China and the United States are undoubtedly the main driving force and in the core position of global research. In the meantime, future researchers should strengthen more extensive cooperation between different countries. Current research directions for CPDT could mainly be divided into four parts including nanomaterial technology, clinical applications, mechanism, and photosensitizers. Of note, “nanotech-based PDT” and “enhanced PDT” were current research hotspots. In the near future, we believe that the promising research directions could concentrate on the following topics: “photothermal therapy,” “nanosphere,” “nanoparticle,” “metal organic framework,” “checkpoint blockade,” “nanomaterial,” “nanoplatform,” “nanomedicine,” “tumor microenvironment,” “gold nanoparticle,” “prodrug.” In a word, this bibliometric study provides a comprehensive analysis of CPDT research, which could provide helpful references for scholars and policymakers in this area.

## Data Availability

The original contributions presented in the study are included in the article/Supplementary Material, further inquiries can be directed to the corresponding authors.
