# Supplementary material for: Bibliometric Analysis of Global Research on Cancer Photodynamic Therapy: Focus on Nano-Related Research
Source: Front Pharmacol. 2022 Jun 16;13:927219. doi: 10.3389/fphar.2022.927219 (PMC9243586; doi:10.3389/fphar.2022.927219)
Supplement: Supplementary file 1 [file DataSheet1.docx]

Supplementary Material

# Supplementary Figures and Tables

## Supplementary Figure


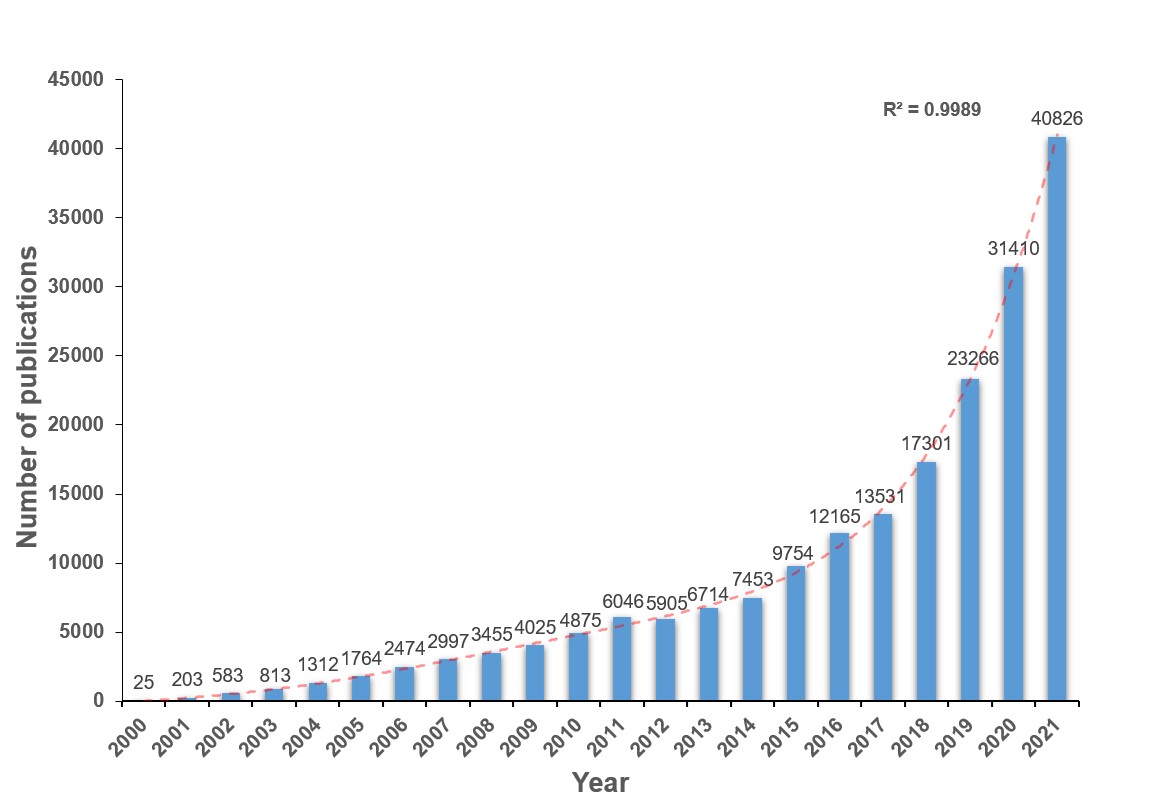


**Supplementary Figure 1.** Distribution of the annual number of citations regarding CPDT research from 2000 to 2021. The blue bars represent the number of papers related to CPDT per year. The red dotted line represents the trend-fitted curve and the correlation coefficients (R^2^) is displayed in the figure.


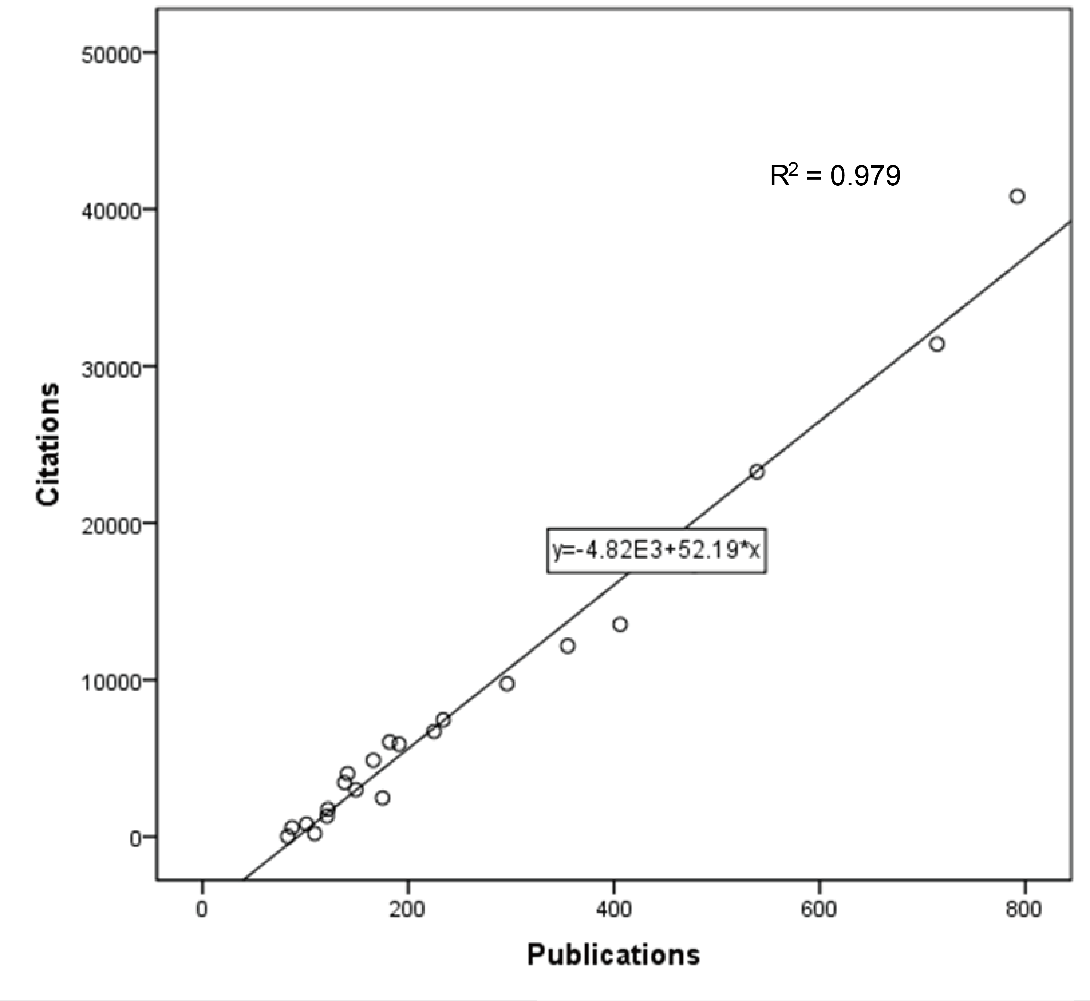


**Supplementary Figure 2.** The correlation between annual publications and citations.


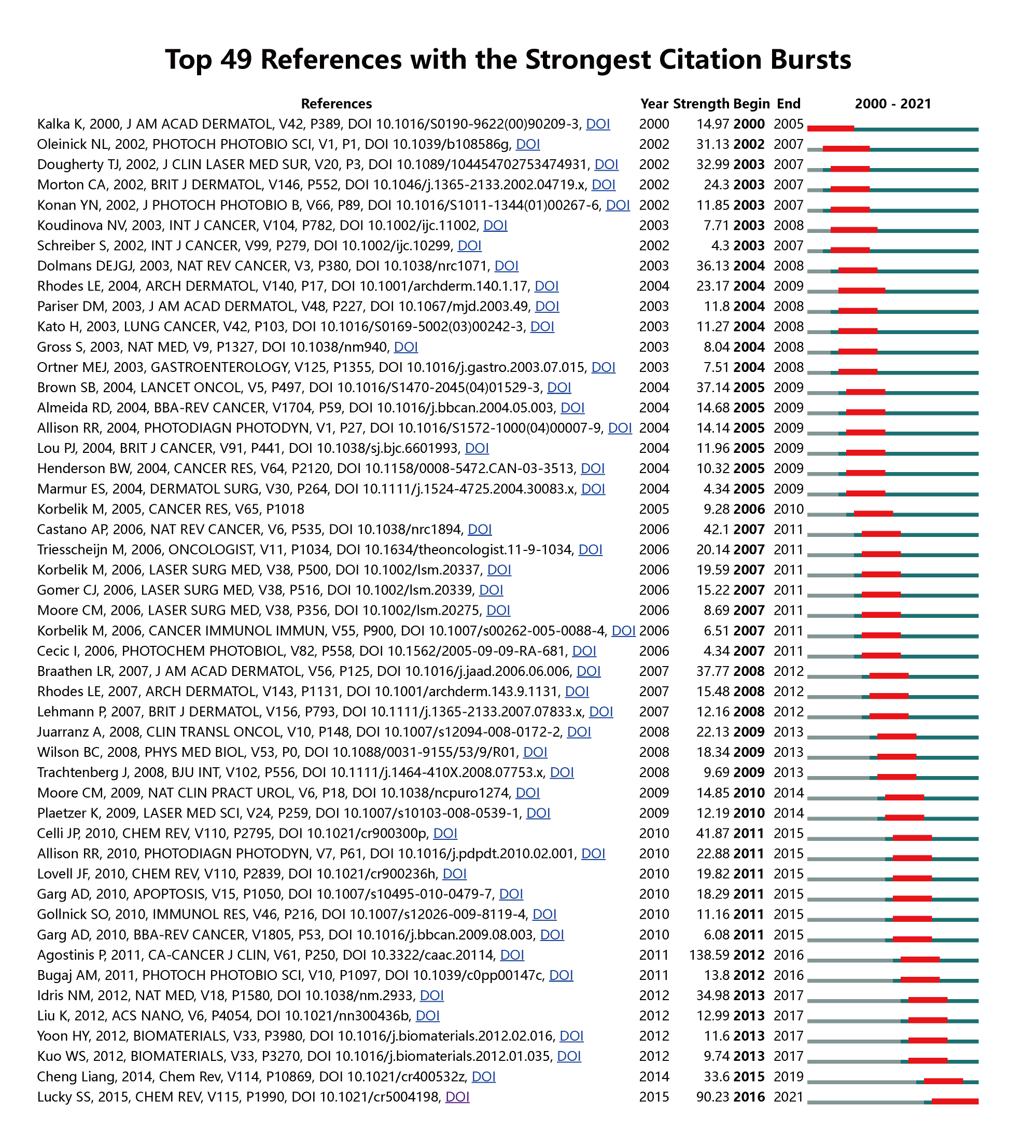


**Supplementary Figure 3.** Top 49 references with the strongest citation bursts by CiteSpace.

**Supplementary Table 1. Top 20 most frequent occurrences keywords**

| **Ranking** | Keywords | occurrences | total link strength | **Ranking** | Keywords | occurrences | total link strength |
| --- | --- | --- | --- | --- | --- | --- | --- |
| **1** | photodynamic therapy | 4262 | 4195 | **11** | cells | 499 | 495 |
| **2** | cancer | 1299 | 1294 | **12** | mechanisms | 432 | 432 |
| **3** | photosensitizers | 1295 | 1294 | **13** | in vivo | 430 | 429 |
| **4** | drug delivery | 1193 | 1192 | **14** | photothermal therapy | 405 | 405 |
| **5** | nanoparticles | 1148 | 1144 | **15** | therapy | 343 | 343 |
| **6** | 5-aminolevulinic acid | 730 | 728 | **16** | chemotherapy | 333 | 331 |
| **7** | apoptosis | 686 | 685 | **17** | porphyrins | 315 | 315 |
| **8** | in vitro | 588 | 587 | **18** | fluorescence | 277 | 275 |
| **9** | tumors | 545 | 541 | **19** | expression | 265 | 265 |
| **10** | singlet oxygen | 501 | 500 | **20** | protoporphyrin ix | 265 | 265 |

**Supplementary Table 2.** **The clusters information of co-cited references**

| Cluster ID | Size | Mean (Year) | Silhouette | Label (LLR algorithm) |
| --- | --- | --- | --- | --- |
| #0 | 44 | 0.971 | 1997 | human epidermoid carcinoma cell |
| #1 | 36 | 0.98 | 2016 | enhanced photodynamic therapy |
| #2 | 35 | 0.971 | 2010 | cancer cell |
| #3 | 35 | 0.874 | 2008 | prostate cancer |
| #4 | 35 | 0.978 | 2010 | clinical use |
| #5 | 30 | 0.987 | 2017 | recent advance |
| #6 | 30 | 0.978 | 2000 | early cancer |
| #7 | 29 | 0.91 | 2001 | tumor stroma |
| #8 | 28 | 0.928 | 2003 | nonmelanoma skin cancer |
| #9 | 27 | 0.951 | 2004 | cellular mechanism |
| #10 | 26 | 0.974 | 1997 | superficial basal cell carcinoma |
| #11 | 21 | 0.939 | 2010 | graphene oxide |
| #12 | 21 | 0.947 | 2002 | cerebral glioma |
| #13 | 20 | 0.929 | 2014 | near infrared light |
| #14 | 18 | 0.839 | 2006 | photodynamic therapy |
| #15 | 17 | 0.932 | 2007 | basal cell carcinoma |
